# Reproductive Schedules in Southern Bluefin Tuna: Are Current Assumptions Appropriate?

**DOI:** 10.1371/journal.pone.0034550

**Published:** 2012-04-13

**Authors:** Karen Evans, Toby A. Patterson, Howard Reid, Shelton J. Harley

**Affiliations:** 1 Wealth from Oceans National Research Flagship, Commonwealth Scientific and Industrial Research Organisation (CSIRO) Division of Marine and Atmospheric Research, Hobart, Tasmania, Australia; 2 New Zealand Ministry of Fisheries, Wellington, New Zealand; Texas A&M University-Corpus Christi, United States of America

## Abstract

Southern bluefin tuna (SBT) appear to comprise a single stock that is assumed to be both mixed across its distribution and having reproductive adults that are obligate, annual spawners. The putative annual migration cycle of mature SBT consists of dispersed foraging at temperate latitudes with migration to a single spawning ground in the tropical eastern Indian Ocean. Spawning migrations have been assumed to target two peaks in spawning activity; one in September-October and a second in February-March. SBT of sizes comparable to that of individuals observed on the spawning ground were satellite tagged in the Tasman Sea region (2003–2008) and demonstrated both migrations to the spawning grounds and residency in the Tasman Sea region throughout the whole year. All individuals undertaking apparent spawning migrations timed their movements to coincide with the second recognised spawning peak or even later. These observations suggest that SBT may demonstrate substantial flexibility in the scheduling of reproductive events and may even not spawn annually as currently assumed. Further, the population on the spawning grounds may be temporally structured in association with foraging regions. These findings provide new perspectives on bluefin population and spatial dynamics and warrant further investigation and consideration of reproductive schedules in this species.

## Introduction

The bluefin tunas (*Thunnus thynnus*, *T. orientalis*, and *T. maccoyii*) are often regarded as the emblematic ocean wanderers amongst teleosts. Able to swim vast distances at high speeds, they are typified by migrations between dispersed, temperate foraging grounds and distinct, tropical spawning grounds, the most extensive of which occur in southern bluefin tuna [1]–[3]. Given the distances between foraging and spawning grounds (which can be on the order of thousands of kilometres), undertaking a spawning migration is an energetically challenging proposition. Time spent in warm, tropical conditions that favour larval thermal requirements may also involve physiological challenges for reproducing individuals. The physiological adaptations toward homeothermy are most advanced in bluefin tunas in comparison to other scombrids [4]–[6]. The cold-adapted physiology of bluefin tunas may result in individuals undergoing significant thermal stress when inhabiting tropical spawning habitats [7], [8] adding to the challenges imposed on individuals associated with migration. As long-lived species (up to 40 yr; [1], [9]–[10]), the life history strategy of bluefin tunas therefore involves trade-offs between energetically costly spawning migrations and expected life-time reproductive output [11]. As a result, it could be expected that reproductive schedules in mature individuals might be somewhat flexible in their timing in response to individual condition. When individual condition is relatively poor, reproductive events might be deferred until individual condition improves to above a certain threshold, thereby maximising lifetime reproductive output [11]. However, empirical data on spawning migrations to confirm or refute such a strategy has been lacking.

Southern bluefin tuna (SBT) is a heavily depleted species (4–11% pre-exploitation biomass; [12]), with a dispersed distribution throughout the temperate waters of the south-eastern Atlantic, south Indian Ocean and south-western Pacific. Although dispersed in distribution, higher concentrations of SBT are found close to the coasts of Argentina, southern Africa, southern Australia and New Zealand, suggesting some geographic structure to the population [9]. Adults forage throughout temperate waters, undertaking extensive migrations to a single identified spawning ground located in the tropical east Indian Ocean [3]. Unlike other bluefin tuna, for which multiple spawning sites have been identified [13]–[15], there is currently little evidence supporting the presence of spawning of SBT in areas outside of the north-east Indian Ocean [16]–[18] and genetic studies conducted to date also suggest a single population [19].

Although spawning SBT are observed on the spawning ground in all months, catch data show two peaks in abundance: one in September-October and a second in February-March, suggesting some degree of synchrony in spawning [18]. Larval survey data also support evidence of an austral spring-summer peak in spawning [16]. Size and age of SBT on the spawning ground (which are presumed to be mature and spawning) have undergone substantial changes over the last decade, decreasing from a mean of 188.1 cm and 19–21 years to 166.8 cm and 14–16 years [20]–[21]. Corresponding increases in growth rates of younger age classes have been observed, most likely as a result of their significant depletion from harvesting [1], [20]–[21]. The most recent data detailing lengths of SBT from the spawning ground demonstrate a mean length of 168.5 cm and 15 years [21].

Size at maturity estimates for the species are based on investigations of gonad weights and histology derived from samples collected from the spawning ground. Such investigations are inherently biased, as they are exclusively derived from the fishery and so therefore do not include samples from outside those areas fished, they are limited to specimens collected from the known spawning ground and they lack inclusion of samples from immature specimens. Size at maturity has therefore not been determined accurately in SBT as yet, but is thought to occur at around 10–12 years and between 152 and 162 cm [22]. Current stock assessment models implicitly assume that SBT comprise a single stock that is completely mixed and individuals are obligate annual spawners once mature. Findings which question any or all of these premises should be considered in association with stock assessment outputs and their use and uptake into management procedures for the fishery.

Development of electronic tags and their implementation in established long-term programs are beginning to provide insights into the migration paths, frequency of movement into areas of spawning, fidelity of fish to foraging regions and mixing of fish between regions for a number of marine fish species [3], [23]–[25]. In this paper, we present electronic tagging data that provide initial support for the conjecture that at least a proportion of the bluefin population might trade off short-term fitness gains from annual spawning against net life-time reproductive advantage via a flexible spawning strategy. We consider the implications of a strategy involving non-annual spawning on current management assumptions for the species.

## Materials and Methods

Pop-up satellite archival tags (PAT2: n = 11, PAT3: n = 8 and PAT4: n = 71, Wildlife Computers, Redmond USA) were deployed on large SBT during the austral winter months in waters off eastern Australia (2001–2006) and northern New Zealand (2007–2008). Tagging methods employed in Australian (AU) waters are detailed in [3] and those employed in New Zealand (NZ) waters were similar to those detailed in [26]. All fish were caught during commercial longline fishing operations. In AU waters, once a fish was caught, it was brought on board the fishing vessel via a purpose made cradle. A length measurement was taken and the satellite tag attached externally via a double anchor system (detailed in [3]) before release back into the water. In NZ waters, fish were brought alongside the fishing vessel, a measurement was estimated and tags were externally attached to individuals whilst in the water via a single anchor system identical to the primary anchor and tether used in AU waters before release of the fish. Prior to release, the hook was either removed from the animal (AU) or the line cut as close to the mouth of the animal as possible (NZ). Sea surface temperatures recorded by vessels at the time of tagging ranged 18.3–21.1°C in AU waters and 13.6–18.3°C in NZ waters. All personnel involved in tagging were experienced in the identification of SBT, the selection of suitable tagging candidates (i.e. those of suitable size and condition) and tagging methods. All efforts were made to ensure tagging was conducted as efficiently as possible while minimizing potential impacts on individuals. The total time of tagging procedures from unclipping of fish from the mainline to release post-tagging was less than two minutes for SBT tagged in the water and less than five minutes for those brought on board vessels. All tagging procedures in AU waters were conducted in strict accordance with Tasmanian Department of Primary Industries, Parks, Water and Environment Animal Ethics Committee approval (permits 3/2003–2004, 23/2006–2007) and under scientific permits issued by the Australian Fisheries Management Authority (AFMA). Tagging activities conducted for management purposes in NZ waters were conducted under provisions in the Sixth Schedule of the New Zealand Fisheries Act (1996).

Tags were programmed to release from fish and transmit summarised depth, temperature and light data after periods of time ranging from 30 days to the limit of the tags, which was 365 days. Full details of tag set-up, proposed and achieved attachment periods and analyses of the movement, habitat and fate of SBT tagged in AU waters are detailed in [3]. Failure of tags to report to the Argos system occurred in 14 of the 90 tags deployed, one of which was later recovered and the data retrieved (03P0357, included in this study). Examination of transmitted data from tags (reports of pin-breakages, maximum depths, depth profile data and summarized depth data) suggests the possibility of mortality in one individual, which occurred 23 days post-deployment. It should be noted that assignment of definite mortality of individuals as apposed to detachment of tags (via disturbance of anchors, tether failure, failure of tag attachment points etc.), is difficult to determine from data transmitted from PSATs due to its summarized nature. Further, if mortality can be identified, assignment of mortality to a causal factor (fishing methods, handling methods, natural environmental mortality) is largely impossible.

**Figure 1:**
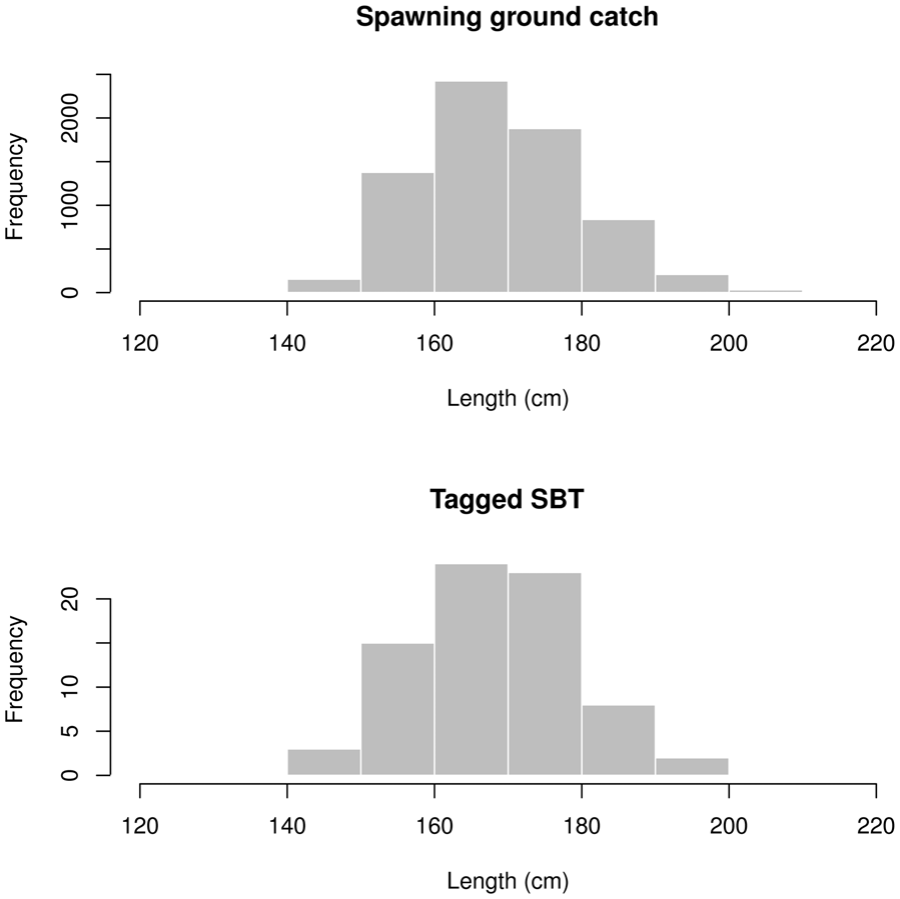
Length frequencies of SBT caught by the Indonesian fishery on the spawning ground and SBT tagged in this study.

In this study, in order to capture at least the initial stages of any spawning migrations, we included only those deployments >160 days in our analyses (Table 1; n = 23). Fish ranged 151–180 cm (length to caudal fork) and closely reflected SBT caught by the Indonesian fishery in the spawning ground area [18] and presumably mature (Figure 1). Using the upper bound of estimates of length at sexual maturity (L_50_) defined in [22], 14 of the 23 SBT for which there were deployments >160 days were larger than 162 cm and therefore could be assumed to be sexually mature. If lower bounds of estimates of L_50_ are used, 21 of the 23 SBT could be assumed to be sexually mature.

**Table 1:** Details of tags deployed in the Tasman Sea 2003–2008, maximum sea surface temperature experienced, spawning classification and actual or estimated month of arrival on the spawning ground.

Tag number	Date deployed	Area deployed	Length (cm lcf)	TAL (days)	Maximum sst (°C)	Classification	Actual/estimated arrival
04P0603	14 Jul 2007	NZ	163	262	29.25	spawner	Feb
03P0350	12 Jul 2004	AU	169	206	28.30	spawner	Feb
04P0343	04 Aug 2006	AU	150	224	21.15	likely spawner	Apr–May
04P0579	04 Jul 2007	NZ	180	199	19.25	likely spawner	Feb–Mar
03P0352	13 Jul 2004	AU	170	185	20.85	likely spawner	Feb–Mar
03P0196	28 Jul 2003	AU	168	180	19.40	likely spawner	Feb–Mar
03P0357	14 Jul 2004	AU	176	172	20.20	likely spawner	Mar–Apr
03P0341	05 Jul 2004	AU	174	165	21.15	likely spawner	Feb–Mar
03P0369	08 Aug 2004	AU	173	160	20.60	likely spawner	Feb–Mar
04P0340	04 Aug 2006	AU	169	233	18.65	likely non-spawner	May–Jun
04P0624	17 Jul 2007	NZ	160	218	20.70	likely non-spawner	May–Jun
04P0346	04 Aug 2006	AU	151	213	18.35	likely non-spawner	May–Jun
04P0344	04 Aug 2006	AU	161	202	20.35	likely non-spawner	May–Jun
04P0476	19 Jul 2008	NZ	175	201	21.35	likely non-spawner	May–Jun
04P0436	08 Aug 2006	AU	167	197	20.55	likely non-spawner	May–Jun
04P0580	12 Jul 2008	NZ	160	185	18.65	likely non-spawner	May–Jun
04P0440	08 Aug 2006	AU	157	182	18.90	likely non-spawner	May–Jun
04P0446	13 Aug 2006	AU	151	182	20.05	likely non-spawner	May–Jun
04P0350	08 Aug 2006	AU	164	172	20.55	likely non-spawner	May–Jun
04P0348	04 Aug 2006	AU	154	170	19.25	likely non-spawner	Apr–May
04P0658	25 Jul 2007	NZ	173	163	17.4	likely non-spawner	May–Jun
04P0626	17 Jul 2007	NZ	160	364	19.20	non-spawner	n/a
04P0607	05 Jul 2007	NZ	167	350	19.65	non-spawner	n/a

AU: Australian waters, NZ New Zealand waters, lcf: length to caudal fork, tal: time at liberty, sst: sea surface temperature.

Daily positions derived from each tag were calculated using the state-space model described in [27] and implemented using the R software package “trackit” (downloaded from: www.soest.hawaii.edu/tag-data/trackit). Movement paths of each tagged fish, satellite transmission locations and the likely duration of migration from the southern Tasman Sea to the spawning grounds (established in [3] to be ∼110 days) were used to classify fish into four categories of migration behaviour (i) definite migration to the spawning ground area (spawners); (ii) movements consistent with spawning migration, but not conclusive due to premature detachment of the tag (likely spawners); (iii) movements consistent with residency in the Tasman Sea, but not conclusive due to premature detachment of the tag (likely non-spawners) and (iv) residency in the Tasman Sea for an annual cycle (non-spawners; we assumed that if a fish had not demonstrated any movement away from known foraging grounds after 350 days, it was unlikely to undertake a spawning migration within that year).

## Results

**Figure 2:**
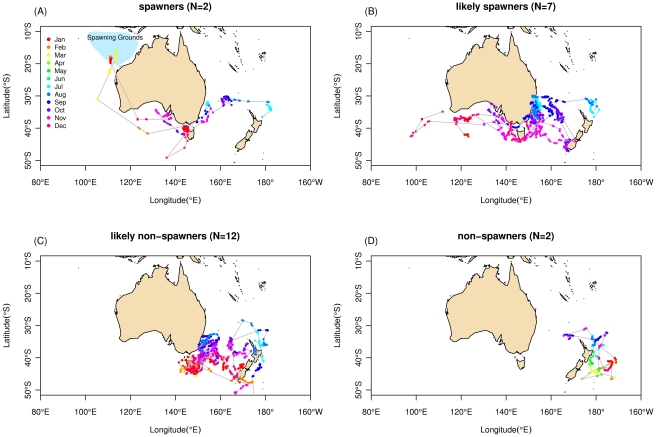
****Movement paths of fish categorized by putative spawning behaviour . (a) spawners showing movements from the tagging region to the spawning grounds (defined in blue) (b) likely spawners which made large westward migrations; (c) likely-non spawners remained in the Tasman Sea region until late in the spawning season and (d) non-spawners which remained resident in the Tasman for a full spawning cycle.

Two SBT (163 and 169 cm) were classified as spawners, arriving on the spawning grounds in February and April (Table 1, Figure 2). A further five SBT (150–180 cm) were classified as likely spawners, undertaking movements towards the south-west Australian region consistent with spawning migration before their tags prematurely detached (Figure 2). Estimates of arrival on the spawning ground of these fish ranged between February and May (Table 1). Two SBT (160 and 167 cm) remained in the Tasman Sea for periods of 364 and 350 days, demonstrating residency in this region throughout the whole year (Table 1, Figure 2) and were classified as non-spawners. A further 12 SBT (151–175 cm) were classified as likely non-spawners, demonstrating movement patterns consistent with residency in the Tasman Sea with no indication of movement consistent with the beginning of a spawning migration (Table 1, Figure 2). If we assume (conservatively) that SBT classified as likely non-spawners had undertaken migrations with a comparable duration to those SBT whose spawning migrations were observed, arrival in the region of the spawning ground was estimated to have occurred between March and June (Table 1).

No SBT other than those that migrated to the spawning ground encountered waters conducive for spawning; all remained in waters with sea surface temperature of less than 21.5°C (Table 1). Size distributions of spawners/likely spawners (mean ± SD: 169.22±8.73) were slightly larger than those of non-spawners/likely non-spawners (mean ± SD: 162.23±7.81), however this was not significant (t-test, d.f. = 15.33, *p* = 0.06) and there was substantial overlap in the distributions of the two groups. One large individual (180 cm), classified as a likely spawner, influenced the *p*-value substantially: removal of this individual from the dataset resulted in a t-test *p*-value of 0.13.

## Discussion

Movement patterns of SBT tagged in the Tasman Sea region are clearly highly variable: individuals likely to be mature undertook spawning migrations, but also remained in the Tasman Sea throughout the year. Spawners and likely spawners were either observed or estimated to arrive on the spawning ground no earlier than February and possibly as late as May. No SBT classified as likely non-spawners were estimated to arrive on the spawning ground earlier than April with the majority estimated to arrive May–June. Only those SBT observed to migrate to the spawning ground encountered waters with sea surface temperatures (SSTs) greater than 24°C and which are considered suitable for spawning [28]. These early data suggest two things: (1) that mature SBT may not spawn on an annual time scale and may exhibit flexibility in the timing of their reproductive schedules and (2) the makeup of SBT on the spawning ground may be related to their choice of winter foraging areas and there is a hitherto unobserved degree of spatial structure in the spawning stock.

Non-annual or temporally flexible spawning behaviour, often termed skipped spawning, is defined as the paired behaviours of failure to migrate and non-spawning [29]. The behaviour is positively correlated with reproductive life span, occurring more frequently in longer-living species, and is generally considered to be evidence of a trade-off between reproduction, growth and survival across the life span of the species [30]. If nutritional or environmental conditions do not meet an individual's energetic cost of migration and gonad development, spawning may not occur. Alternatively, if conditions are good, resources may be invested in accelerated growth rather than spawning, particularly in younger age classes of mature fish, as attaining a larger size may actually result in increased fecundity in subsequent years [30].

Non-annual or skipped spawning has been recorded in several marine fish, including Atlantic cod (*Gadus morhua*; [31]), Atlantic herring (*Clupea harengus*; [32]), blue grenadier (*Macruronus novaezelandiae*; [33]), striped bass (*Morone saxatilis*; [34]) and orange roughy (*Hoplostethus atlanticus*; [35]). Amongst the tunas, similar behaviour has not been conclusively documented to date. However, catch data and a number of tagging studies have reported adult Atlantic bluefin tuna and Pacific bluefin tuna in waters away from spawning areas during the spawning season [36]–[40], suggesting the possibility that fish either spawn outside of known spawning regions, or that spawning occurs across temporal periods other than that previously observed for these species [10], [40].

Age and length at maturity in SBT are considered to be approximately 10–12 years and 152–162 cm [18], [22], [41] with individuals as young as 6 years and 145 cm caught on the spawning ground [42]. Examination of the gonads of females sampled from the spawning ground reported all females to be mature [18], and so it is reasonable to assume that fish can mature at sizes as small as 145 cm. While we cannot conclusively determine whether or not fish included in this study were mature, SBT observed to undertake spawning migrations and those remaining resident in the Tasman Sea were of similar sizes to current estimates of length at maturity in SBT. All SBT were also of sizes reflective of those caught on the spawning ground.

Spawning in SBT is reported to occur in waters where surface water temperatures are greater than 24°C [1], [10], [28]. The requirement to spawn in warm waters is largely associated with the physiological requirements of larvae for optimal feeding and growth, as they are not yet endothermic [43]. Pacific bluefin tuna have been observed to spawn in captivity in waters of surface temperatures of 21.6–29.2 [44]. In instances where individuals were observed to spawn at temperatures below 24°C, water temperatures in the days prior to spawning had been at least 23°C before dropping on the day of spawning, suggesting that individuals may require a degree of temporal consistency in temperatures >23°C for spawning, and that spawning is not necessarily associated with consistently lower temperatures. Examination of growth rates of SBT larvae on the spawning ground in the eastern Indian Ocean recorded larvae of four to 13 days old occurring in temperatures 27.25–27.70°C [45]. Temperatures recorded were considered to be representative of those experienced by larvae and in association, waters in which spawning occurs due to the consistency of temperatures in the upper mixed layer in the region [45]. Maximum SSTs recorded from tagged individuals in this study ranged 17.40°C to 29.25°C, with only those individuals that migrated to the spawning ground region experiencing temperatures that are consistent with current understanding of suitable temperatures for spawning of SBT, that is, temperatures >24°C. All other individuals recorded maximum SSTs of 21.35°C or less. Based on assumptions of suitable spawning habitat and maximum SSTs recorded by SBT, it is unlikely that individuals could have been spawning in areas outside the known spawning ground and in particular, in the Tasman Sea.

Recent modelling studies of Atlantic bluefin tuna examining energy allocation and the decision to spawn found that, given the cost of migration, young mature bluefin were likely to defer spawning, and that this optimised life long fecundity [46]. While evidence to confirm this reproductive strategy is yet to be observed, it may be that such a reproductive strategy is common amongst all bluefin and is also present in SBT. This behaviour is hypothesised to decline as fish age due to the larger size of older mature fish which are more often in better condition and for which less energy expenditure is required for growth [46].

Temporal variation of SBT abundance on the spawning ground has also been documented elsewhere. It has been postulated that this variability might be associated with the dispersed distribution of foraging adult SBT throughout the Southern Ocean in the winter months [18]. Migration distances and time taken to reach the spawning ground of SBT will vary with winter foraging locations. If initiation of spawning migration occurs at the same time of the year through the whole population, those individuals foraging closer to the spawning ground (e.g. Indian Ocean) will reach the spawning grounds earlier in the season than those foraging further away (e.g. Tasman Sea).

The triggers for initiation of migration in SBT are currently unknown. Temperature-associated variation in spawning migration timing has been documented in a number of fish species [47]–[48] and has been linked with gonadal development, requirements to time spawning with seasonal peaks in productivity [35] and water temperatures conducive to larval development and survival [49]. Variability in temperature gradients across the Southern Ocean foraging ground for SBT may drive differences in the onset of migration to the spawning ground dependent on where a fish is foraging during the winter months. This may also be the reason why the spawning season is protracted in comparison to spawning in the other bluefin species. In the Tasman Sea region, ocean temperature variability associated with the Tasman Front and East Australia Current (EAC) might influence the onset of migration and result in the initiation of migration not occurring until later in the year, in comparison to the Indian Ocean region. This would result in SBT foraging in the Tasman Sea not contributing to the spawning population until the second half of the spawning season. Inter-annual variability in water temperatures across the Southern Ocean and in the location of frontal features and boundary currents such as the Tasman Front and EAC may also affect the onset of migration and could be responsible for shifts observed in peaks in abundance of SBT on the spawning ground between years [18].

It could be argued that potential negative impacts associated with tagging procedures may influence the timing of migration and/or if large enough, actual migration itself. The deployment of tags on animals is, in itself, a disturbing process with impacts on individuals varying with capture methods, types of tags, attachment methods and length of deployments [50]. A number of authors have noted that diving behaviour in marine animals on which PSATs have been deployed, has been altered at least for an initial few hours post-tagging [51]–[52]. Beyond these initial few hours post-tagging, differences reported in vertical diving behaviour have been noted to become hard to discern from the rest of the time series of diving data collected after release These observations suggest, at least in these fish, that any marked behavioural changes associated with the deployment of PSATs have potentially been short-term. Visual inspection of archival records of diving behaviour from recovered PSATs deployed on SBT tagged in the Tasman Sea (n = 9; 14–213 days at liberty) also demonstrate little quantifiable evidence of long-term altered diving behaviour post release ([3]; CSIRO unpublished data). It must be noted however, that smaller scale changes in diving behaviour are not able to be determined from summarised data transmitted from PSATs and archival records required to investigate these changes are often lacking from PSATs (as retrieval of the archival record requires recovery of the tag). Further, details of non-reporting of tags, attachment periods programmed (as opposed to attachment periods achieved) and potential mortalities are often lacking from the data [3]. Finally, all tagging studies, regardless of the technology and the attachment method used are limited by the simple fact that it is impossible to record the behaviour of untagged fish against which the behaviour of tagged individuals can be compared. Therefore, all assessments of impacts of the deployment of tags on individuals are often based on sparse data, which may contain unknown biases.

Despite these problems, electronic tagging has recorded many instances of apparently normal behaviour. Atlantic bluefin tuna tagged with archival tags, which have been surgically implanted into the peritoneal cavity, have demonstrated continued migration to spawning grounds in the Gulf of Mexico in the year of tagging and have been observed entering the Gulf of Mexico at the same time each year over multiple subsequent years [53]. The maintenance of continued migration in the year of tagging and migration in the years following tagging suggests no significant or long-term impact of internal placement of tags on the onset of migration. Extending the length of datasets collected from PSATs across multiple years would allow the collection of similar multi-year datasets and examination of potential changes in the occurrence and onset of migration. Recent development of PSATs utilising solar power sources, in addition to traditional battery power sources, may facilitate such longer-term deployments and assessments by extending the life of tags. Alternatively, similar deployments of internally implanted archival tags would facilitate the collection of multi-year data. This however, incurs substantive cost, as large numbers of tags are required to be deployed in order to return reasonable dataset sizes. Further, determining the movement patterns of species using archival tags, is subject to a number of biases associated with reliance of recapture and return of archival tags for data recovery, including potential differential temporal and spatial effort and differential reporting rates across species distributions.

The extent to which flexible reproductive schedules affect annual spawning events has potential implications for stock assessments and rebuilding of the population. If flexible reproductive schedules have occurred historically at a consistent level within the population, their impact on annual spawning stock biomass, egg production and recruitment should be more or less constant. However, if the proportion of the population or particular age classes delaying or skipping annual spawning is variable or has undergone a consistent change, it may impact on population productivity. This effect is likely to be further enhanced at low population levels, such as those currently experienced by the SBT population.

This study has provided early indications of greater variability in spawning behaviour than is implicitly assumed by current population models for SBT. Without a definitive measure of maturity and condition for tagged SBT however, it is difficult to conclusively identify reasons as to why some SBT did not migrate toward the spawning ground and how this behaviour might vary with age and size. Multi-year records of movement (e.g. [53]) would provide greater insights into the potential for skipped spawning to occur; the frequency it might occur at, and any association with particular age classes (e.g. younger, mature individuals). They would also be useful for examining variation in spawning migration, initiation and how these might be related to potential triggers such as ocean temperature.
